# C-Peptide: A Mediator of the Association Between Serum Uric Acid to Creatinine Ratio and Non-Alcoholic Fatty Liver Disease in a Chinese Population With Normal Serum Uric Acid Levels

**DOI:** 10.3389/fendo.2020.600472

**Published:** 2020-11-19

**Authors:** Chifa Ma, Yiwen Liu, Shuli He, Jingbo Zeng, Pingping Li, Chunxiao Ma, Fan Ping, Huabing Zhang, Lingling Xu, Wei Li, Yuxiu Li

**Affiliations:** ^1^ Department of Endocrinology, Key Laboratory of Endocrinology, Ministry of Health, Peking Union Medical College Hospital, Peking Union Medical College, Chinese Academy of Medical Sciences, Beijing, China; ^2^ Department of Nutrition, Peking Union Medical College Hospital, Beijing, China; ^3^ Department of Endocrinology, Fuxing Hospital, the Eighth Clinical Medical College, Capital Medical University, Beijing, China; ^4^ State Key Laboratory of Bioactive Substance and Function of Natural Medicines, Institute of Materia Medica, Chinese Academy of Medical Sciences and Peking Union Medical College, Beijing, China; ^5^ Diabetes Research Center of Chinese Academy of Medical Sciences, Beijing, China

**Keywords:** C‐peptide (blood), mediated effect, non-alcoholic fatty liver disease, normal-ranged uric acid, serum uric acid to creatinine ratio

## Abstract

**Background:**

The data on the relationship between normal-ranged serum uric acid (SUA), β-cell function, and non-alcoholic fatty liver disease (NAFLD) are complicated and insufficient. Moreover, uric acid is excreted by kidney, and SUA levels may be affected by renal function. Thus, we introduced a renal function-normalized index [serum uric acid to creatinine ratio (SUA/Cr)] into the study and explored the association between SUA/Cr, C‐peptide and NAFLD in a Chinese population with normal SUA levels by a cross-sectional analysis.

**Materials and Methods:**

A total of 282 individuals with normal SUA levels and different glucose tolerance status from a diabetes project were included in the study (mean age = 53.7± 10.5 years; women = 64.50%). NAFLD was diagnosed by abdominal ultrasonography (NAFLD, n=86; without NAFLD, n=196). Trapezoid formula was used to calculate area under the curve of C‐peptide (AUC_CP_) from 4 points (including 0, 30,60, and 120min) during 2-h oral glucose tolerance test. Spearman correlation analysis was used to explore the correlation between SUA/Cr, AUC_CP_ and NAFLD risk factors. Multiple logistic regression analysis was used to explore the association between SUA/Cr or AUC_CP_ and NAFLD. Mediation analysis was used to explore whether AUC_CP_ mediated the association between SUA/Cr and NAFLD.

**Results:**

Individuals with NAFLD had significantly higher SUA/Cr and AUC_CP_ than those without NAFLD(P<0.05). Spearman correlation analysis showed that both SUA/Cr and AUC_CP_ were significantly associated with many NAFLD risk factors, and SUA/Cr was positively correlated with AUC_CP_ (P<0.05). Multiple logistic regression analysis indicated that SUA/Cr and AUC_CP_ were positively associated with NAFLD incidence (P<0.05). Medication analysis indicated that SUA/Cr had a significant direct effect on NAFLD (β =0.5854, 95% CI: 0.3232–0.8966), and AUC_CP_ partly mediated the indirect effect of SUA/Cr on NAFLD incidence (β =0.1311, 95% CI: 0.0168–0.4663).

**Conclusions:**

SUA/Cr was positively associated with NAFLD incidence, and AUC_CP_ partly mediated the association in a Chinese population with normal SUA levels. Thus, we should pay more attention to high-normal SUA and C-peptide levels due to their predictive power in NAFLD incidence.

## Introduction

Non-alcoholic fatty liver disease (NAFLD), characterized by lipid accumulation in liver with no significant alcohol intake, is one of the most common chronic liver diseases in the world. NAFLD can develop into cirrhosis, and even hepatocellular carcinoma and liver failure (1).NAFLD is also closely associated with cardiovascular disease (2), diabetes (3), and obesity (4).Moreover, patients with NAFLD exhibited relative high mortality compared to the general population (5). Hence, finding risk factors and the mechanism of NAFLD is warranted to prevent it.

Serum uric acid (SUA), major product of purine metabolism, is independently associated with NAFLD incidence (6, 7). Moreover, the significant association between SUA and NAFLD incidence was also established even in some individuals with normal SUA levels (8, 9). SUA is excreted *via* kidney, and the clearance of SUA is often affected by renal function, while none of the previous studies (6–9) considered the effects from kidney. SUA to creatinine ratio (SUA/Cr) is an index of renal function-normalized SUA, reflecting endogenous UA levels more precisely than SUA. Moreover, SUA/Cr is associated with β-cell function, metabolic syndrome and incident chronic kidney disease (10–12).However, there has been no study focused on the association between SUA/Cr and NAFLD yet.

In addition to NAFLD, SUA was also associated with islet β-cell function and β-cell secretion in patients with type 2 diabetes (T2DM) and prediabetes (13, 14). Moreover, SUA, within normal range, was related to β-cell function in overweight/obesity or male T2DM patients (15). Although the causal relationship between SUA and β-cell function was not conclusive, one study indicated that elevated SUA was the precursor of T2DM (16).There were also some studies reporting the significant association between β-cell secretion and NAFLD. An American study indicated that fasting C-peptide (FCP) was associated with NAFLD (17). A Chinese study based on obese children also found that FCP is a significant indicator of NAFLD (18). C-peptide and the area under the curve of C‐peptide (AUC_CP_) could reflect the secretion ability of islet β-cell, while the latter is a better indicator of overall and residual islet β-cell secretion compared to FCP. However, the studies involving the association between AUC_CP_ and NAFLD are limited.

Several clinical studies have indicated that increased SUA, within the normal range, is closely associated with many diseases (19, 20), and high-normal SUA(elevated SUA within normal range) has already hold our attention. The intrinsic relationship between SUA, β-cell function and NAFLD is complicated, and related studies involving normal-ranged SUA are insufficient. Moreover, renal function may be also a potential confounding factor of the association between SUA and NAFLD. Thus, we performed a cross-sectional study based on a Chinese population with normal SUA levels and introduced an index of renal function-normalized SUA into the study, namely, SUA/Cr, and explored the association between SUA/Cr, AUC_CP_, and NAFLD.

## Materials and Methods

### Study Population

**Figure 1:**
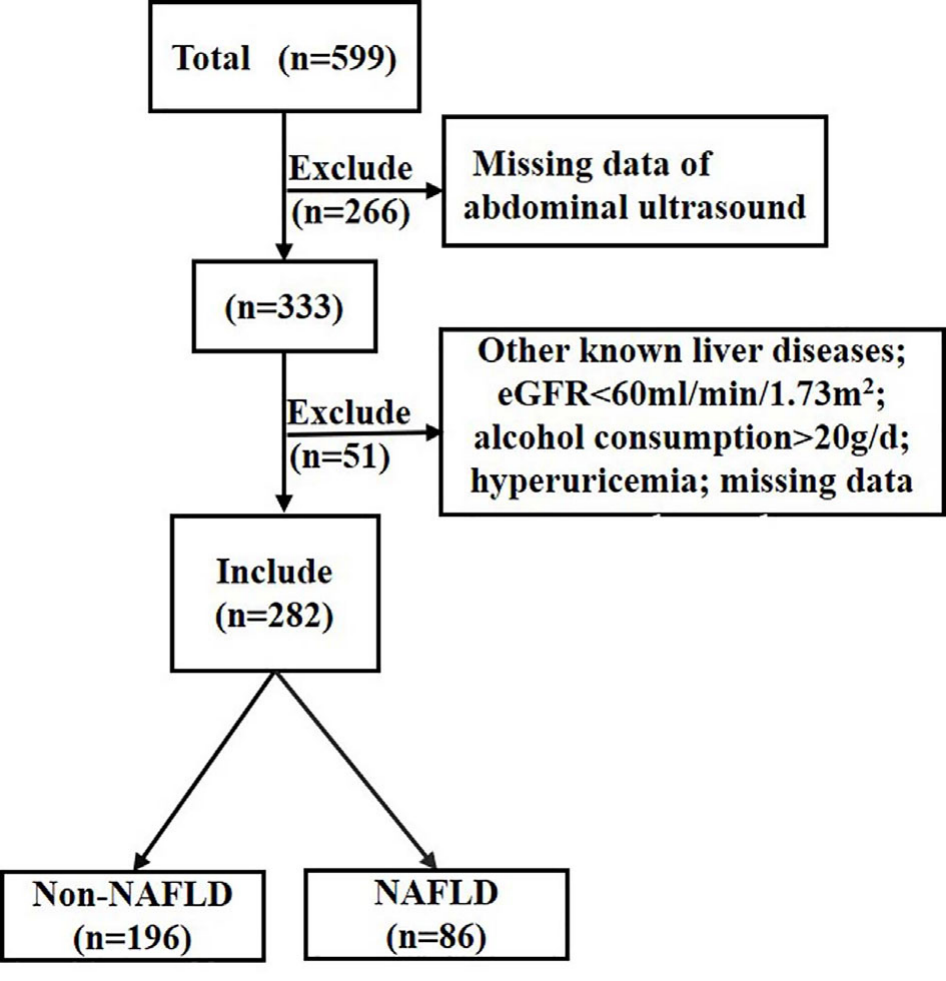
Flow chart of the population inclusion. NAFLD, non-alcoholic fatty liver disease; eGFR, estimated glomerular filtration rate. eGFR was calculated with the chronic kidney disease epidemiology collaboration equation.

A total of 599 individuals from a T2DM project were recruited between 2014 and 2015 (21), and 333 of them completed liver ultrasound. Participants with other liver diseases, estimated glomerular filtration rate <60 ml/min/1.73 m^2^, alcohol consumption >20 g/day, hyperuricemia (SUA ≥ 416 µmol/L for male or SUA ≥ 357 umol/L for female or uric acid-lowering drugs treatment) or missing data were also excluded (n=51). A total of 282 individuals with normal SUA levels and with different glucose tolerance status were included in the study (**Figure 1**).

### Anthropometric and Biochemical Measurements

All participants were asked to complete a questionnaire, including gender, age, and medical history. Blood pressure, waist circumference (WC) and body mass index (BMI) were measured using standard methods. Blood samples for glucose and C-peptide were obtained at 0, 30, 60, and 120 min after a 75-g oral glucose load. Serum C peptide levels were measured by chemiluminescence immunoassay using a Siemens ADIVA Centaur XP analyzer (Siemens Healthcare Diagnostics Inc., Tarrytown, NY, USA), while serum glucose concentrations were assayed using a glucose oxidase assay. Hemoglobin A1c (HbA1c) analysis was analyzed in whole blood using high-performance liquid chromatography. The diagnosis of diabetes and prediabetes were defined based on the1999 World Health Organization criteria after the oral glucose tolerance test (OGTT) (22). Fasting serum alanine transaminase (ALT), aspartate transaminase (AST), SUA, creatinine, and lipids were measured by an automated analyzer. Trapezoid formula was used to calculate AUC for glucose and C-peptide. Insulin resistance and insulin sensitivity was estimated using homeostasis model assessment of insulin resistance (HOMA-IR) (23).

### Liver Ultrasonography Evaluation

NAFLD was diagnosed by abdominal ultrasonography. The ultrasound results were assessed by physicians who were blind to the subjects’ biochemical results. Except for individuals with significant alcohol consumption, subjects were diagnosed with NAFLD by ultrasonography if at least two of the following three ultrasonic characteristics were positive: bright liver, liver echo greater than kidney, vascular blurring, and deep attenuation of ultrasound signal (24).

### Statistical Analysis

Normally distributed continuous variables were recorded as mean ± standard deviation. Non-normal distribution parameters were transformed or presented as the median (25th–75th percentile). Categorical data were presented as number and percentages (n, %). Differences between groups were compared by Student’s t test or Chi-squared test or Mann–Whitney’s U-test. Spearman correlation analysis was used to explore the association between SUA/Cr or AUC_CP_ and potential NAFLD risk factors. Multiple logistic regression analysis was used to evaluate the association between SUA/Cr or AUC_CP_ and NAFLD. Mediation models were established to explore whether AUC_CP_ mediated the association between SUA/Cr and NAFLD.

Analyses were conducted using the SPSS (version 22.0). *P* < 0.05 (2-tailed) was considered statistically significant.

## Results

### Clinical Characteristics of Individuals in NAFLD and Non-NAFLD Groups

According to the liver ultrasonography, individuals were divided into a NAFLD group (n=86) and a non-NAFLD group (n=196). As recorded in **Table 1**, individuals with NAFLD had higher BMI, WC, total cholesterol (TC), triglycerides (TG), low- density lipoprotein cholesterol (LDL-C), HbA1c, 2-h post-load glucose(2hPG), area under the curve of glucose (AUC_Glu_), FCP, 2-h post-load C-peptide (2hCP), AUC_CP_, HOMA-IR, ALT, AST, and SUA/Cr but lower high-density lipoprotein cholesterol (HDL-C) compared to those without NAFLD(P<0.05). In addition, individuals with NAFLD were more likely to have higher prevalence of diabetes than those without NAFLD (**Table 1**).

**Table 1:** Characteristics of the NAFLD and non-NAFLD groups in individuals with normal uric acid.

	Non-NAFLD	NAFLD	P
n	196	86	
Gender			0.224
Female, n (%)	122(62.2)	60(69.8)	
Male, n (%)	74(37.8)	26(30.2)	
Age, years	53.8 ± 11.2	53.5 ± 8.8	0.799
BMI, kg/m^2^	24.65 ± 2.92	28.42 ± 3.83	<0.001
WC, cm	84.89 ± 8.61	92.27 ± 9.60	<0.001
SBP, mmHg	128.15 ± 19.89	129.83 ± 17.75	0.503
DBP, mmHg	74.61 ± 9.78	76.59 ± 9.96	0.121
TC, mmol/L	5.43 ± 1.07	5.71 ± 1.09	0.045
LnTG, mmol/L	0.22 ± 0.52	0.61 ± 0.54	<0.001
HDL-C, mmol/L	1.34 ± 0.39	1.21 ± 0.22	0.002
LDL-C, mmol/L	2.77 ± 0.74	3.08 ± 0.71	0.001
SUA/Cr	3.90 ± 0.89	4.56 ± 1.51	<0.001
FBG, mmol/L	5.95(5.38-6.66)	6.09(5.54-7.24)	0.054
2h PG, mmol/L	7.23(5.89-8.45)	8.15(6.33-11.72)	0.003
AUC_Glu_	1050.38(865.58-1284.38)	1161.45(962.25-1595.96)	0.006
FCP, ng/ml	1.15(0.86-1.40)	1.65(1.28-2.20)	<0.001
2hCP, ng/ml	4.80(3.52-6.32)	6.71(4.34-8.55)	<0.001
AUC_CP_	541.35(440.06-690.79)	659.25(510.56-868.39)	<0.001
HbA1c, %	5.5(5.2-5.8)	5.7(5.45-6.30)	0.001
LnHOMA-IR	0.81 ± 0.64	1.28 ± 0.58	<0.001
ALT, U/L	21.1(16.5-27.9)	28.1(20.9-42.6)	<0.001
AST, U/L	21.8(18.6-25.1)	23.0(20.2-28.6)	0.021
Glucose tolerance status			0.013
Diabetes, n (%)	41(20.9)	30(34.9)	
Prediabetes, n (%)	63(32.1)	30(34.9)	
NGT, n (%)	92(46.9)	26(30.2)	

Data were presented as mean ± standard deviation, number (percentage), or median (25th–75th percentile). NAFLD, non-alcoholic fatty liver disease; BMI, body mass index; WC, waist circumference; SBP, systolic blood pressure; DBP, diastolic blood pressure; TC, total cholesterol; TG, triglycerides; HDL-C, high-density lipoprotein cholesterol; LDL-C, low-density lipoprotein cholesterol; SUA/Cr, serum uric acid to creatinine ratio; FBG, fasting blood glucose; 2h PG, 2-h post-load glucose; AUC_Glu_, area under the curve of glucose; FCP, fasting C-peptide; 2hCP, 2-h post-load C-peptide; AUC_CP_, area under the curve of C-peptide; HbA1c, hemoglobin A1c; HOMA-IR, homeostatic model assessment of insulin resistance; ALT, alanine aminotransferase; AST, aspartate aminotransferase; NGT, normal glucose tolerance.

### Spearman Correlation of SUA/Cr or AUC_CP_ With Potential NAFLD Risk Factors

As shown in **Table 2**, Spearman correlation analysis indicated that SUA/Cr was positively correlated with BMI (r=0.208, P<0.001),WC (r=0.217, P<0.001), TG (r=0.285, P<0.001), LDL-C (r=0.151, P=0.011), HbA1c(r=0.120, P=0.045), HOMA-IR(r=0.198, P=0.001), ALT(r=0.190, P=0.001), AST(r=0.183, P=0.002) and AUC_Glu_ (r=0.124, P=0.037) but negatively correlated with HDL-C (r=-0.176, P=0.003). Similarly, AUC_CP_ was positively correlated with BMI (r=0.256, P<0.001),WC (r=0.197, P=0.001), TG (r=0.295, P<0.001), LDL-C (r=0.137, P=0.022), HOMA-IR(r=0.455, P<0.001), ALT(r=0.159, P=0.007), and AST(r=0.117, P=0.049) but negatively correlated with HDL-C(r=-0.251, P<0.001).There were also significant correlations between SUA/Cr and C-peptide related markers [FCP(r=0.246, P<0.001), 2hCP(r=0.190, P=0.001), AUC_CP_ (r=0.208, P<0.001)].

**Table 2:** Spearman’s correlation of SUA/Cr or AUC_CP_ with potential risk factors of non-alcoholic fatty liver disease.

	SUA/Cr		AUC_CP_	
	r	P	r	P
Age	0.115	0.053	0.046	0.446
BMI	0.208	<0.001	0.256	<0.001
WC	0.217	<0.001	0.197	0.001
SBP	0.094	0.114	0.077	0.199
DBP	0.017	0.781	-0.033	0.578
TC	0.101	0.092	0.052	0.383
Ln TG	0.285	<0.001	0.295	<0.001
HDL-C	-0.176	0.003	-0.251	<0.001
LDL-C	0.151	0.011	0.137	0.022
HbA1c	0.120	0.045	-0.101	0.091
LnHOMA-IR	0.198	0.001	0.455	<0.001
ALT	0.190	0.001	0.159	0.007
AST	0.183	0.002	0.117	0.049
FBG	0.087	0.147	-0.066	0.271
2hPG	0.103	0.084	-0.044	0.462
AUC_Glu_	0.124	0.037	-0.021	0.730
FCP	0.246	<0.001	_	_
2hCP	0.190	<0.001	_	_
AUC_CP_	0.208	<0.001	_	_

SUA/Cr, serum uric acid to creatinine ratio; AUC_CP_, area under the curve of C-peptide; BMI, body mass index; WC, waist circumference; SBP, systolic blood pressure; DBP, diastolic blood pressure; TC, total cholesterol; TG, triglycerides; HDL-C, high-density lipoprotein cholesterol; LDL-C, low-density lipoprotein cholesterol; HbA1c, hemoglobin A1c; HOMA-IR, homeostatic model assessment of insulin resistance; ALT, alanine aminotransferase; AST, aspartate aminotransferase; FBG, fasting blood glucose; 2hPG, 2-h post-load glucose; AUC_Glu_, area under the curve of glucose; FCP, fasting C-peptide; 2hCP, 2-h post-load C-peptide.

### Association Between SUA/Cr and the Prevalence of NAFLD

As shown in **Table 3**, multiple logistic analysis indicated SUA/Cr was positively associated with the prevalence of NAFLD [Odds ratio (OR): 2.288, 95% confidence intervals (CI): 1.592–3.288, P<0.001] after adjustment for age and gender (Model 1). After further adjustment for BMI, WC, systolic blood pressure (SBP), TG, HDL-C, LDL-C, HOMA-IR, and glucose tolerance status, the association between SUA/Cr and NAFLD incidence remained significant (OR: 1.529, 95% CI: 1.011-2.310, P=0.044, Model 2). In addition, SUA/Cr still showed a significant association with NAFLD incidence after additional adjustment for ALT and AST (OR: 1.548, 95% CI: 1.018–2.352, P=0.041, Model 3).

**Table 3:** Logistic regression analysis for association of SUA/Cr with non-alcoholic fatty liver disease.

SUA/Cr	OR (95%CI)	P
Model 1	2.288(1.592,3.288)	<0.001
Model 2	1.529(1.011,2.310)	0.044
Model 3	1.548(1.018,2.352)	0.041

Model 1 was adjusted for age and gender. Model 2 was adjusted for the covariates of model 1 plus body mass index, waist circumference, systolic blood pressure, triglycerides, high-density lipoprotein cholesterol, low-density lipoprotein cholesterol, homeostasis model assessment of insulin resistance, and glucose tolerance status. Model 3 was adjusted for the covariates of model 2 plus alanine transaminase and aspartate transaminase. Odds ratios and 95% CIs were calculated per 1-SD increment of SUA/Cr. SUA/Cr, serum uric acid to creatinine ratio; OR, odds ratio; CI, confidence interval.

### Association Between AUC_CP_ and the Prevalence of NAFLD

As shown in **Table 4**, multiple logistic analysis indicated a positive association between AUC_CP_ and the prevalence of NAFLD (OR: 5.649 95% CI: 2.666-11.971, P<0.001) after adjustment for age and gender (Model 1). After further adjustment for BMI, WC, SBP, TG, HDL-C, LDL-C, HOMA-IR, and glucose tolerance status, the association between AUC_CP_ and NAFLD incidence remained significant (OR: 3.074, 95% CI: 1.166–8.105, P=0.023, Model 2). SUA/Cr still showed a significant association with NAFLD after additional adjustment for ALT and AST (OR: 2.763, 95% CI: 1.012–7.544, P=0.047, Model 3).

**Table 4:** Logistic regression analysis for association of AUC_CP_ with non-alcoholic fatty liver disease.

AUC_CP_	OR (95%CI)	P
Model 1	5.649(2.666,11.971)	<0.001
Model 2	3.074(1.166,8.105)	0.023
Model 3	2.763(1.012,7.544)	0.047

Model 1 was adjusted for age and gender. Model 2 was adjusted for the covariates of model 1 plus body mass index, waist circumference, systolic blood pressure, triglycerides, high-density lipoprotein cholesterol, low-density lipoprotein cholesterol, homeostasis model assessment of insulin resistance, and glucose tolerance status. Model 3 was adjusted for the covariates of model 2 plus alanine transaminase and aspartate transaminase. Odds ratios and 95% CIs were calculated per 1-SD increment of AUC_CP._ AUC_CP_, area under the curve of C-peptide; OR, odds ratio; CI, confidence interval.

### Mediated Effect of AUC_CP_ on the Association Between SUA/Cr and NAFLD

Both SUA/Cr and AUC_CP_ were positively associated with NAFLD incidence, while SUA/Cr was positively correlated with AUC_CP_, suggesting a mechanistic link between SUA/Cr and NAFLD, possibly explained by AUC_CP_. To explore the internal relationships between AUC_CP_, SUA/Cr and NAFLD, we conducted mediation analysis to explore whether AUC_CP_ mediated the association between SUA/Cr and NAFLD incidence.

**Figure 2:**
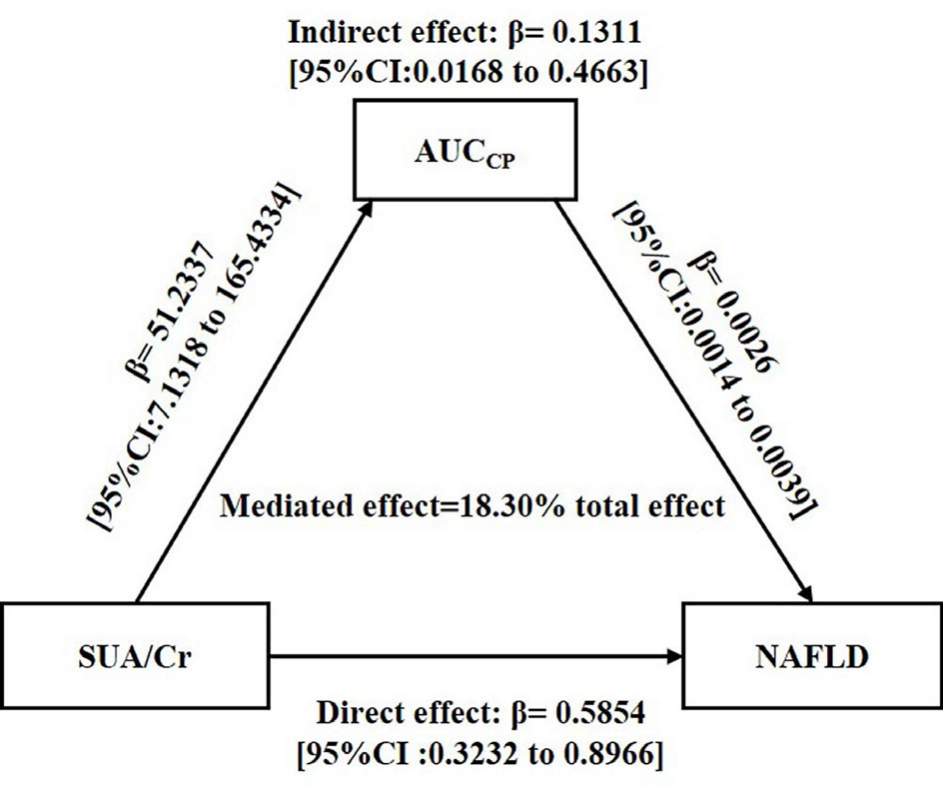
Mediation of AUC_CP_ on the association between SUA/Cr and NAFLD. Zero was not included in 95% confidence intervals representing statistical significance. SUA/Cr, serum uric acid to creatinine ratio; AUC_CP_, area under the curve of C-peptide; NAFLD, non-alcoholic fatty liver disease.

As shown in **Figure 2**, mediation analysis indicated that SUA/Cr had a significant direct effect on NAFLD incidence (β =0.5854, 95% CI: 0.3232–0.8966), and AUC_CP_ partly mediated the indirect effect of SUA/Cr on NAFLD incidence (β =0.1311, 95% CI: 0.0168–0.4663).

## Discussion

This study, based on a Chinese population with normal SUA levels, indicated that individuals with NAFLD had higher SUA/Cr and AUC_CP_ than those without NAFLD. Both SUA/Cr and AUC_CP_ were significantly correlated with many conventional risk factors of NAFLD, and the correlation between SUA/Cr and AUC_CP_ was positive. In addition, SUA/Cr was positively associated with NAFLD incidence, and AUC_CP_ partly mediated the indirect effect of SUA/Cr on NAFLD incidence in a population with normal SUA levels.

The association between SUA and NAFLD has been explored for a long time. A Chinese study contained 21,798 subjects revealed that SUA was significantly associated with NAFLD incidence (25). A prospective observational study demonstrated that high SUA independently predicted 3-year’s incidence of NAFLD (26). Moreover, even in individuals with normal SUA levels, increased SUA was independently associated with NAFLD (8, 9). UA is the end product of human purine metabolism and is excreted by the kidney. SUA level will increase due to its impaired clearance in individuals with impaired renal function (27).However, many studies ignored the effect of kidney on SUA, while SUA/Cr is a renal function-normalized index and may be a more precise indicator than SUA. A Chinese study based on 713 diabetics revealed that SUA/Cr was significantly associated with β-cell function (11). Another study suggested that SUA/Cr in T2DM patients was closely linked to metabolic syndrome and its components (10). Furthermore, a longitudinal study indicated that SUA/Cr had stronger associations with chronic kidney disease than SUA alone (12). Similarly, our present study firstly demonstrated that SUA/Cr was an independent risk factor of NAFLD in individuals with normal SUA levels, and mediation analysis indicated SUA/Cr had direct effect on NAFLD. Although the detailed mechanism of NAFLD remains uncertain, many studies have indicated that there is a close association between SUA and NAFLD. SUA may function as a pro-oxidant and react with oxidants, inducing the production of free radicals and oxidative stress (28), which are critical factors in the development of NAFLD (29).Thus, SUA may have direct effect on NAFLD as a pro-oxidant.

We also found the effect of SUA/Cr on NAFLD partly *via* AUC_CP_. Several studies have indicated that FCP was independently associated with NAFLD (17, 18),while we used a more accurate index (AUC_CP_), which could reflect overall β-cell secretion. Equimolar amount of C-peptide is produced when insulin is secreted. However, insulin, not C-peptide, is partly cleared in liver with first-pass hepatic extraction (30). Thus, serum C-peptide was a well-established marker of the endogenous insulin secretion. We found that individuals with NAFLD had higher AUC_CP_ and HOMA-IR, and AUC_CP_ was significantly correlated with HOMA-IR. We also found AUC_CP_ partly mediated the association between SUA/Cr and NAFLD. Although the causal relationship between UA and insulin resistance was not conclusive, elevated SUA may aggravate insulin resistance to some extent. A clinical study indicated that elevated SUA was the precursor of T2DM (16). SUA could induce endothelial dysfunction and inhibit nitric oxide bioavailability, which is involved in insulin resistance (31). Thus, higher AUC_CP_ may represent higher endogenous insulin secretion and may be a compensatory of insulin resistance due to higher SUA, namely, higher AUC_CP_ is a sign of insulin resistance, which plays important role in the progress of NAFLD (32). In other hand, many metabolic regulators such as follistatin and fibroblast growth factor (FGF21) were regulated by islet hormone (33, 34). Moreover, fold changes of C-peptide during an OGTT were inversely associated with those of FGF21 in individuals with normal glucose tolerance (35), and FGF21 related signal pathways played important roles in the progression of NAFLD (36). In addition, previous studies have indicated that C-peptide may be also a predictive marker of the severity of the cardiovascular disease (37) and mortality (38), which suggested C-peptide was a bioactive peptide with other potential physiological functions. Thus, AUC_CP_ may partly mediate the association between SUA/Cr and NAFLD *via* insulin resistance and other potential physiological function.

Besides AUC_CP_, other mechanism may be also involved in the association between SUA and NAFLD. A previous study indicated that SUA, within normal range, was positively associated with inflammation markers (39), which may be the important mediator in the development of NAFLD (40). Basic studies showed that SUA also caused hepatic steatosis and liver fat accumulation *via* endoplasmic reticulum stress (41) and mitochondrial oxidative stress (42).In addition, SUA may generate from fructose metabolism, which could induce hepatic steatosis (43). Overall, high-normal SUA was positively associated with NAFLD incidence *via* its direct pro-oxidant effect, C-peptide, and other signal pathways. Thus, high-normal SUA and C-peptide levels are important factors in the pathological process of NAFLD, and we should pay enough attention to these indicators.

Our present study had some advantages. First, present study introduced SUA/Cr as a newer index into the study and revealed that there was a significant association between SUA/Cr and NAFLD incidence, which brought a more accurate predictor of NAFLD. Second, we explored the internal relationship between SUA/Cr, AUC_CP_, and NAFLD by different statistical methods, which strengthens our understanding of their internal relationship. Third, our present study based on a population with normal SUA levels and found its strong predictive ability for NAFLD, which suggested that high-normal SUA should cause our attention. Our resent study also had some limitations. First, liver biopsy has been established as the gold diagnosis standard of NAFLD, while NAFLD was determined by ultrasonography with no histologic confirmation in our present study. Nevertheless, ultrasonography is the widely-used methodology to detect NAFLD because of safety, availability, and economy. Second, the nature of the cross-sectional study and the relatively small sample size were also the limitations of the present study, and therefore larger scale and longitude studies are warranted in the future.

## Conclusions

SUA/Cr was positively associated with NAFLD incidence, and AUC_CP_ partly mediated the association between SUA/Cr and NAFLD incidence in a Chinese population with normal SUA levels. This finding indicates that we should pay more attention to high-normal SUA and C-peptide levels due to their predictive power in NAFLD incidence.

## Data Availability Statement

All datasets presented in this study are included in the article/supplementary material.

## Ethics Statement 

The studies involving human participants were reviewed and approved by the Ethics Committee of Peking Union Medical College Hospital. The patients/participants provided their written informed consent to participate in this study.

## Author Contributions

CFM conducted the research, performed the statistical analysis, and wrote the first draft of the manuscript. YiL, SH, JZ, PL, CXM, FP, HZ, LX, and WL contributed to the discussion, conducted the research, and collected the data. YuL designed the study and revised the manuscript. All authors contributed to the article and approved the submitted version.

## Funding

This project was supported by the CAMS Innovation Fund for Medical Sciences (CIFMS) (CIFMS2016-I2M-4-001) and the Non-profit Central Research Institute Fund of Chinese Academy of Medical Sciences (No. 2017PT32020, No. 2018PT32001, and No. 2019PT320007).

## Conflict of Interest

The authors declare that the research was conducted in the absence of any commercial or financial relationships that could be construed as a potential conflict of interest.

## Abbreviations

ALT, alanine transaminase; AST, aspartate transaminase; AUC_CP_, area under the curve of C‐peptide; AUC_Glu_, area under the curve of glucose; BMI, body mass index; CI, confidence interval; FCP, fasting C-peptide; 2hCP, 2-h post-load C-peptide; FGF21, fibroblast growth factor 21; HDL-C, high-density lipoprotein cholesterol; HOMA-IR, homeostasis model assessment of insulin resistance; 2hPG, 2-h post-load glucose; LDL-C, low-density lipoprotein cholesterol, NAFLD, non-alcoholic fatty liver disease; OR, odds ratio; SUA, serum uric acid; SUA/Cr, serum uric acid to creatinine ratio; TC, total cholesterol; T2DM, type 2 diabetes; TG, triglycerides; WC, waist circumference.

## References

[1] Serfaty L Lemoine M Definition and natural history of metabolic steatosis: clinical aspects of NAFLD, NASH and cirrhosis. Diabetes Metab (2008) 34(6 Pt 2):634–7. 10.1016/S1262-3636(08)74597-X 19195623

[2] Targher G Day CP Bonora E Risk of cardiovascular disease in patients with nonalcoholic fatty liver disease. N Engl J Med (2010) 363(14):1341–50. 10.1056/NEJMra0912063 20879883

[3] Tilg H Moschen AR Roden M NAFLD and diabetes mellitus. Nat Rev Gastroenterol Hepatol (2017) 14(1):32–42. 10.1038/nrgastro.2016.147 27729660

[4] Fan JG Kim SU Wong VW New trends on obesity and NAFLD in Asia. J Hepatol (2017) 67(4):862–73. 10.1016/j.jhep.2017.06.003 28642059

[5] Ekstedt M Hagstrom H Nasr P Fredrikson M Stal P Kechagias S Fibrosis stage is the strongest predictor for disease-specific mortality in NAFLD after up to 33 years of follow-up. Hepatology (2015) 61(5):1547–54. 10.1002/hep.27368 25125077

[6] Li Y Xu C Yu C Xu L Miao M Association of serum uric acid level with non-alcoholic fatty liver disease: a cross-sectional study. J Hepatol (2009) 50(5):1029–34. 10.1016/j.jhep.2008.11.021 19299029

[7] Lee JW Cho YK Ryan M Kim H Lee SW Chang E Serum uric Acid as a predictor for the development of nonalcoholic Fatty liver disease in apparently healthy subjects: a 5-year retrospective cohort study. Gut Liver (2010) 4(3):378–83. 10.5009/gnl.2010.4.3.378 PMC2956352 20981217

[8] Hwang IC Suh SY Suh AR Ahn HY The relationship between normal serum uric acid and nonalcoholic fatty liver disease. J Korean Med Sci (2011) 26(3):386–91. 10.3346/jkms.2011.26.3.386 PMC3051086 21394307

[9] Moon SS Relationship between serum uric acid level and nonalcoholic fatty liver disease in pre- and postmenopausal women. Ann Nutr Metab (2013) 62(2):158–63. 10.1159/000346202 23406781

[10] Al-Daghri NM Al-Attas OS Wani K Sabico S Alokail MS Serum Uric Acid to Creatinine Ratio and Risk of Metabolic Syndrome in Saudi Type 2 Diabetic Patients. Sci Rep (2017) 7(1):12104. 10.1038/s41598-017-12085-0 28935934 PMC5608718

[11] Li M Gu L Yang J Lou Q Serum uric acid to creatinine ratio correlates with beta-cell function in type 2 diabetes. Diabetes Metab Res Rev (2018) 34(5):e3001. 10.1002/dmrr.3001 29504280

[12] Gu L Huang L Wu H Lou Q Bian R Serum uric acid to creatinine ratio: A predictor of incident chronic kidney disease in type 2 diabetes mellitus patients with preserved kidney function. Diabetes Vasc Dis Res (2017) 14(3):221–5. 10.1177/1479164116680318 28183204

[13] Tang W Fu Q Zhang Q Sun M Gao Y Liu X The association between serum uric acid and residual beta -cell function in type 2 diabetes. J Diabetes Res (2014) 2014:709691. 10.1155/2014/709691 24971368 PMC4058242

[14] Wu Y He H Yu K Zhang M An Z Huang H The Association between Serum Uric Acid Levels and Insulin Resistance and Secretion in Prediabetes Mellitus: A Cross-Sectional Study. Ann Clin Lab Sci (2019) 49(2):218–23. 31028067

[15] Zhong X Zhang D Yang L Du Y Pan T The relationship between serum uric acid within the normal range and beta-cell function in Chinese patients with type 2 diabetes: differences by body mass index and gender. PeerJ (2019) 7:e6666. 10.7717/peerj.6666 30941277 PMC6440459

[16] Juraschek SP McAdams-Demarco M Miller ER Gelber AC Maynard JW Pankow JS Temporal relationship between uric acid concentration and risk of diabetes in a community-based study population. Am J Epidemiol (2014) 179(6):684–91. 10.1093/aje/kwt320 PMC3939847 24418684

[17] Atsawarungruangkit A Chenbhanich J Dickstein G C-peptide as a key risk factor for non-alcoholic fatty liver disease in the United States population. World J Gastroenterol (2018) 24(32):3663–70. 10.3748/wjg.v24.i32.3663 PMC6113719 30166861

[18] Han X Xu P Zhou J Liu Y Xu H Fasting C-peptide is a significant indicator of nonalcoholic fatty liver disease in obese children. Diabetes Res Clin Pract (2020) 160:108027. 10.1016/j.diabres.2020.108027 31958476

[19] Ray L Mohandas K Shamraj M Akila B eds. Association of high-normal serum uric acid levels with abnormal lipid profile and high atherogenic index of plasma in apparently healthy South Indian males. Tirupati, India: Ambicon (2014).

[20] Jung DH Lee YJ Lee HR Lee JH Shim JY Association of renal manifestations with serum uric acid in Korean adults with normal uric acid levels. J Korean Med Sci (2010) 25(12):1766–70. 10.3346/jkms.2010.25.12.1766 PMC2995231 21165292

[21] Zhou MC Zhu L Cui X Feng L Zhao X He S Reduced peripheral blood mtDNA content is associated with impaired glucose-stimulated islet beta cell function in a Chinese population with different degrees of glucose tolerance. Diabetes Metab Res Rev (2016) 32(7):768–74. 10.1002/dmrr.2814 PMC5108437 27103506

[22] Alberti KG Zimmet PZ Definition, diagnosis and classification of diabetes mellitus and its complications. Part 1: diagnosis and classification of diabetes mellitus provisional report of a WHO consultation. Diabet Med (1998) 15: (7):539. 10.1002/(SICI)1096-9136(199807)15:7<539::AID-DIA668>3.0.CO;2-S 9686693

[23] Matthews DR Hosker JP Rudenski AS Naylor BA Treacher DF Turner RC Homeostasis model assessment: insulin resistance and beta-cell function from fasting plasma glucose and insulin concentrations in man. Diabetologia (1985) 28(7):412–9. 10.1007/bf00280883 3899825

[24] Fan JG Jia JD Li YM Wang BY Lu LG Shi JP Guidelines for the diagnosis and management of nonalcoholic fatty liver disease: update 2010. J Dig Dis (2011) 12(1):38–44. 10.1111/j.1751-2980.2010.00476.x. published in Chinese on Chinese Journal of Hepatology 2010; 18:163–166. 21276207

[25] Liang J Pei Y Gong Y Liu XK Dou LJ Zou CY Serum uric acid and non-alcoholic fatty liver disease in non-hypertensive Chinese adults: the Cardiometabolic Risk in Chinese (CRC) study. Eur Rev Med Pharmacol Sci (2015) 19(2):305–11. 25683947

[26] Xu C Yu C Xu L Miao M Li Y High serum uric acid increases the risk for nonalcoholic Fatty liver disease: a prospective observational study. PloS One (2010) 5(7):e11578. 10.1371/journal.pone.0011578 20644649 PMC2904389

[27] Johnson RJ Kang DH Feig D Kivlighn S Kanellis J Watanabe S Is there a pathogenetic role for uric acid in hypertension and cardiovascular and renal disease? Hypertension (2003) 41(6):1183–90. 10.1161/01.HYP.0000069700.62727.C5 12707287

[28] Sautin YY Imaram W Kim KM Angerhofer A Henderson G Johnson R Uric Acid and Oxidative Stress. In: Miyata T, Eckardt K-U, Nangaku M, editors. Studies on Renal Disorders. Totowa, NJ: Humana Press (2011). p. 143–59.

[29] Spahis S Delvin E Borys JM Levy E Oxidative Stress as a Critical Factor in Nonalcoholic Fatty Liver Disease Pathogenesis. Antioxid Redox Signal (2017) 26(10):519–41. 10.1089/ars.2016.6776 27452109

[30] Polonsky KS Rubenstein AH C-peptide as a measure of the secretion and hepatic extraction of insulin. Pitfalls and limitations. Diabetes (1984) 33(5):486–94. 10.2337/diab.33.5.486 6373457

[31] Li C Hsieh MC Chang SJ Metabolic syndrome, diabetes, and hyperuricemia. Curr Opin Rheumatol (2013) 25(2):210–6. 10.1097/BOR.0b013e32835d951e 23370374

[32] Akhtar DH Iqbal U Vazquez-Montesino LM Dennis BB Ahmed A Pathogenesis of Insulin Resistance and Atherogenic Dyslipidemia in Nonalcoholic Fatty Liver Disease. J Clin Transl Hepatol (2019) 7(4):362–70. 10.14218/JCTH.2019.00028 PMC6943204 31915606

[33] Hansen JS Rutti S Arous C Clemmesen JO Secher NH Drescher A Circulating Follistatin Is Liver-Derived and Regulated by the Glucagon-to-Insulin Ratio. J Clin Endocrinol Metab (2016) 101(2):550–60. 10.1210/jc.2015-3668 26652766

[34] Hansen JS Clemmesen JO Secher NH Hoene M Drescher A Weigert C Glucagon-to-insulin ratio is pivotal for splanchnic regulation of FGF-21 in humans. Mol Metab (2015) 4(8):551–60. 10.1016/j.molmet.2015.06.001 PMC4529499 26266087

[35] Lin Z Gong Q Wu C Yu J Lu T Pan X Dynamic change of serum FGF21 levels in response to glucose challenge in human. J Clin Endocrinol Metab (2012) 97(7):E1224–8. 10.1210/jc.2012-1132 22539584

[36] Tucker B Li H Long X Rye KA Ong KL Fibroblast growth factor 21 in non-alcoholic fatty liver disease. Metabolism (2019) 101:153994. 10.1016/j.metabol.2019.153994 31672443

[37] Harnishsingh B Rama B Is C-peptide a predictor of severity of coronary artery disease in metabolic syndrome? An observational study. Indian Heart J (2018) 70(Suppl 3):S105–S9. 10.1016/j.ihj.2018.07.005 PMC6309290 30595240

[38] Min JY Min KB Serum C-peptide levels and risk of death among adults without diabetes mellitus. CMAJ (2013) 185(9):E402–8. 10.1503/cmaj.121950 PMC3680586 23589428

[39] Ruggiero C Cherubini A Ble A Bos AJ Maggio M Dixit VD Uric acid and inflammatory markers. Eur Heart J (2006) 27(10):1174–81. 10.1093/eurheartj/ehi879 PMC2668163 16611671

[40] Stojsavljevic S Gomercic Palcic M Virovic Jukic L Smircic Duvnjak L Duvnjak M Adipokines and proinflammatory cytokines, the key mediators in the pathogenesis of nonalcoholic fatty liver disease. World J Gastroenterol (2014) 20(48):18070–91. 10.3748/wjg.v20.i48.18070 PMC4277948 25561778

[41] Choi YJ Shin HS Choi HS Park JW Jo I Oh ES Uric acid induces fat accumulation via generation of endoplasmic reticulum stress and SREBP-1c activation in hepatocytes. Lab Invest (2014) 94(10):1114–25. 10.1038/labinvest.2014.98 25111690

[42] Lanaspa MA Sanchez-Lozada LG Choi YJ Cicerchi C Kanbay M Roncal-Jimenez CA Uric acid induces hepatic steatosis by generation of mitochondrial oxidative stress: potential role in fructose-dependent and -independent fatty liver. J Biol Chem (2012) 287(48):40732–44. 10.1074/jbc.M112.399899 PMC3504786 23035112

[43] Ackerman Z Oron-Herman M Grozovski M Rosenthal T Pappo O Link G Fructose-induced fatty liver disease: hepatic effects of blood pressure and plasma triglyceride reduction. Hypertension (2005) 45(5):1012–8. 10.1161/01.HYP.0000164570.20420.67 15824194

